# Sevoflurane Versus Propofol for Postoperative Sedation After Cardiac Surgery: A Systematic Review of Randomized Controlled Trials

**DOI:** 10.7759/cureus.108341

**Published:** 2026-05-06

**Authors:** Majed M Madkhali, Abdulrahman Y Safhi, Nawaf A Khormi, Sarah H Shok, Afnan H Abuzawah, Thamer A Al-Shehri, Shatha K Alharbi, Mays A Muafa, Abdulrahman Y Alfifa, Majed A Alhamli, Bader H Alqahtani, Yazeed S Almohammadi, Dalal M Alhuian, Amal M Alhuian

**Affiliations:** 1 Anesthesia, Prince Muhammed bin Nasser Hospital, Jazan, SAU; 2 Anesthesia, Jazan Specialized Hospital, Jazan, SAU; 3 College of Medicine, Jazan University, Jazan, SAU; 4 Faculty of Medicine, Jazan University, Jazan, SAU; 5 College of Medicine, King Faisal University, Hofuf, SAU; 6 College of Medicine, Alexandria University, Alexandria, EGY; 7 College of Medicine, King Khalid University, Abha, SAU; 8 Faculty of Medicine, Taibah University, Al-Madinah Al-Munawwarah, SAU; 9 College of Medicine, University of Bisha, Bisha, SAU; 10 Nursing, Cardiac Care Unit (CCU), Riyadh First Health Cluster - King Saud Medical City, Riyadh, SAU

**Keywords:** cardiac surgery, icu sedation, intensive care unit, length of stay, mechanical ventilation, propofol, randomized controlled trials, sevoflurane, systematic review, time to extubation

## Abstract

Optimal postoperative sedation after cardiac surgery remains debated. Sevoflurane delivered via anesthetic conserving devices has emerged as an alternative to intravenous (IV) propofol, but its comparative effectiveness and safety in the intensive care unit (ICU) remain uncertain. This systematic review and meta-analysis evaluated the efficacy and safety of sevoflurane versus propofol for postoperative ICU sedation after cardiac surgery.

We conducted the review in accordance with Preferred Reporting Items for Systematic Reviews and Meta-Analyses (PRISMA) guidelines and the Cochrane Handbook. Medline, Web of Science, Scopus, and CENTRAL were searched without date restrictions. Randomized controlled trials (RCTs) comparing inhaled sevoflurane with IV propofol in mechanically ventilated adult patients admitted to the ICU after cardiac surgery were included. Primary outcomes were time to extubation, ICU length of stay (LOS), and hospital LOS. Secondary outcomes included acute renal failure (ARF), atrial fibrillation, mortality, and postoperative nausea and vomiting (PONV).

Eight RCTs involving 688 patients were included. Four studies reporting time to extubation showed no significant difference between groups overall (mean difference (MD) -62.67, 95% confidence interval (CI) -157.23 to 31.90; p = 0.2977), with high heterogeneity (I² = 91.7%). Sensitivity analysis excluding one study showed significantly shorter extubation time with sevoflurane (MD −72.51, 95% CI -135.83 to -9.20; p = 0.0388). No significant differences were observed for ICU LOS (MD -0.29, 95% CI -4.10 to 3.51) or hospital LOS (MD -0.40, 95% CI -2.67 to 1.63). Secondary outcomes, including mortality, ARF, atrial fibrillation, and PONV, were also comparable between groups.

Sevoflurane appears comparable to propofol for postoperative sedation after cardiac surgery and may reduce time to extubation in selected analyses; however, the evidence remains limited by heterogeneity and possible publication bias.

## Introduction and background

Analgesics and sedatives are routinely administered to patients in the intensive care unit (ICU) after cardiac surgery to improve tolerance of mechanical ventilation and to manage pain and anxiety [1-4]. However, prolonged sedation is associated with extended ICU stays and increased risks of delirium, mortality, and postoperative cognitive dysfunction [2,3]. These risks may be reduced by promoting earlier and more predictable awakening, allowing for frequent and reliable neurological assessments.

Commonly used ICU sedatives include intravenous (IV) benzodiazepines, such as midazolam and lorazepam, and propofol, often combined with opioids or dexmedetomidine [1,5]. These agents are frequently associated with adverse effects, including oversedation, prolonged mechanical ventilation, and hypotension, which can contribute to longer ICU stays [6-8]. In addition, their metabolism and clearance depend on adequate hepatic and renal function, posing challenges in older cardiac surgery patients, who have a higher prevalence of organ dysfunction. Propofol remains widely used due to its rapid onset, short duration of action, and effectiveness in achieving deep sedation [3,5].

Inhalational anesthetics have emerged as an alternative for sedation in mechanically ventilated ICU patients. Volatile agents such as sevoflurane, desflurane, and isoflurane have favorable pharmacokinetic profiles and may facilitate faster awakening, improved neurological recovery, and shorter time to extubation [6,8]. They may also confer cardioprotective effects through mechanisms such as ischemic preconditioning and postconditioning, which can reduce myocardial ischemia-reperfusion injury.

Sevoflurane, a halogenated inhalational anesthetic, offers advantages over other volatile agents, including rapid onset and clearance and a lower incidence of adverse effects [2,5]. When administered using an Anaesthetic Conserving Device (AnaConDa), it provides a practical option for ICU sedation. It has been proposed as an alternative to IV propofol for short-term sedation after cardiac procedures such as coronary artery bypass grafting (CABG). This study aims to evaluate the efficacy and safety of sevoflurane compared with propofol for postoperative ICU sedation in patients undergoing cardiac surgery.

## Review

Methods

Study Design and Reporting

This systematic review and meta-analysis was conducted in accordance with the Preferred Reporting Items for Systematic Reviews and Meta-Analyses (PRISMA) guidelines [9].

Literature Search Strategy

A comprehensive search was performed across four electronic databases: Medline, Web of Science (WOS), Scopus, and the Cochrane Central Register of Controlled Trials (CENTRAL). The search included the keywords “sevoflurane,” “propofol,” and “cardiac surgery,” with no restrictions on publication date. Duplicates were removed using EndNote software. Forward and backward citation screening of included studies and relevant reviews was conducted to identify additional eligible studies.

Eligibility Criteria and Study Selection

Studies were selected based on the Population, Intervention, Comparator, and Outcome (PICO) framework [10]. The population included critically ill adult patients (≥18 years) admitted to the ICU after cardiac surgery, including CABG, valve procedures, off-pump surgery, or combined procedures, who required mechanical ventilation and sedation.

The intervention was inhaled sevoflurane administered via an AnaConDa or Mirus reflector, with any sedation duration or target depth measured by validated scales such as the bispectral index (BIS) or Richmond Agitation-Sedation Scale (RASS) [11]. The comparator was IV propofol, administered as a continuous infusion, with or without adjunct analgesia. Only randomized controlled trials (RCTs) were included.

Data Extraction

Two reviewers independently extracted data using a standardized spreadsheet. Extracted data included study characteristics (e.g., country, design, type of surgery, sedation protocol), baseline patient characteristics (e.g., age, sex, body mass index (BMI), comorbidities, operative variables), and outcomes.

Primary outcomes were time to extubation, ICU length of stay (LOS), and hospital LOS. Secondary outcomes included acute renal failure (ARF), atrial fibrillation or arrhythmia, mortality, and postoperative nausea and vomiting (PONV).

Quality Assessment

The risk of bias of included studies was independently assessed by two reviewers using the Cochrane Risk of Bias 2 (ROB2) tool [12], with disagreements resolved by a third reviewer. The assessment covered randomization, deviations from intended interventions, missing outcome data, outcome measurement, and selective reporting. Each domain was rated as low risk, some concerns, or high risk of bias.

Statistical Analysis

Statistical analysis was performed using R software (version 4.4; R Foundation for Statistical Computing, Vienna, Austria). Mean differences (MDs) were calculated for continuous outcomes and odds ratios (ORs) for dichotomous outcomes, each with 95% confidence intervals (CIs). Statistical significance was set at p < 0.05.

Heterogeneity was assessed using the I² statistic and corresponding p-values, with I² < 40% considered low, 40%-60% moderate, and > 60% high heterogeneity. Sensitivity analyses and Galbraith plots were used to explore heterogeneity and assess robustness. Subgroup analyses were conducted for primary outcomes based on the type of surgery.

Results

Literature Search Results

**Figure 1:**
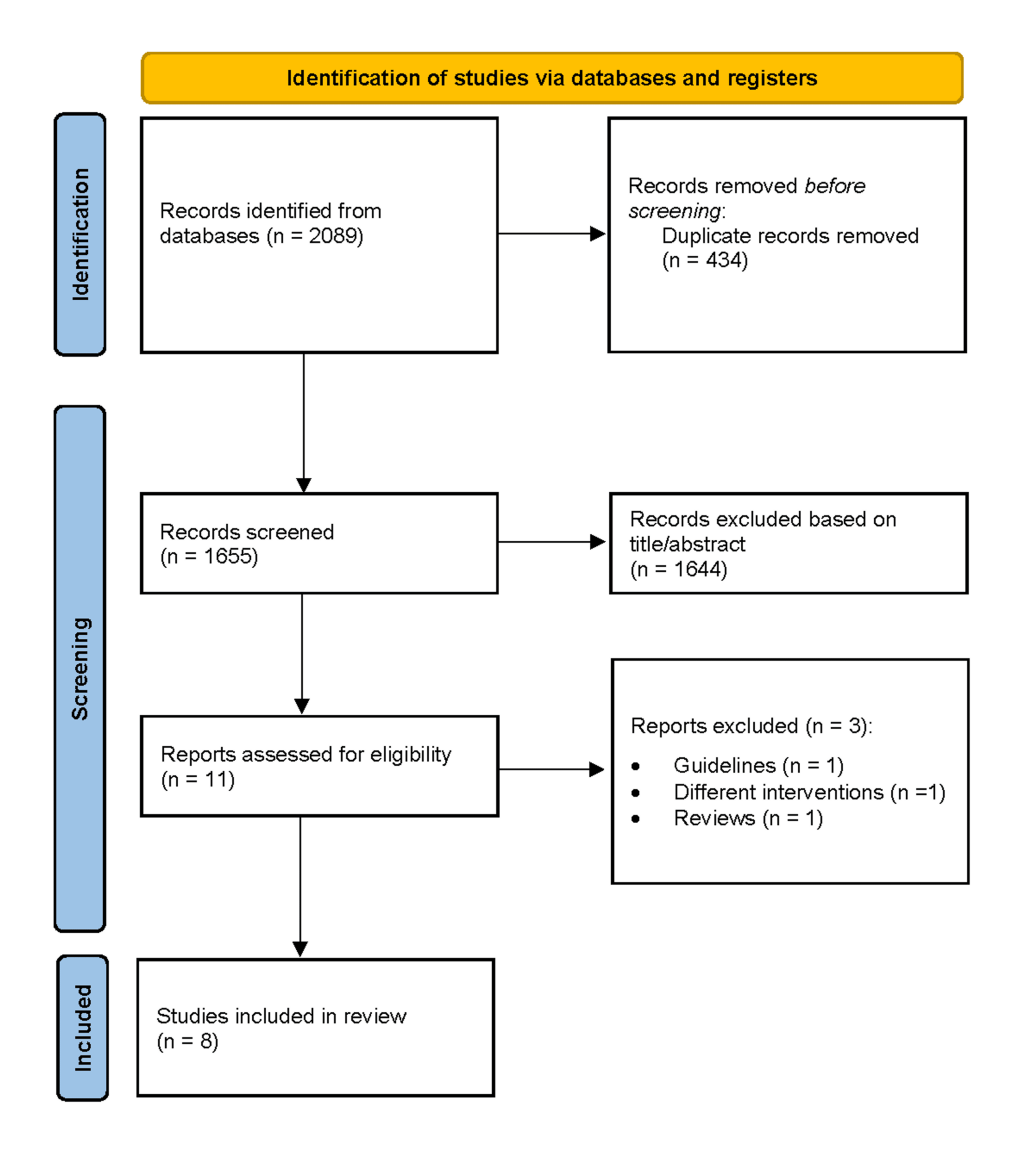
PRISMA flow diagram depicting the study selection process for the systematic review PRISMA (Preferred Reporting Items for Systematic Reviews and Meta-Analyses) flow diagram detailing the study selection process for this systematic review [9]. Following the identification of records through database searching and other sources, duplicates were removed, and the remaining records were screened. Full-text articles were assessed for eligibility, with exclusions documented along with reasons. Studies meeting the inclusion criteria were included in the final synthesis.

The database search identified 2,098 records, of which 1,655 remained after duplicate removal. Following title, abstract, and full-text screening, eight RCTs [1-8] were included in the meta-analysis (Figure 1).

Study Characteristics and Quality Assessment

Eight RCTs [1-8], published between 2008 and 2024 and including a total of 688 patients, compared sevoflurane with propofol for postoperative sedation after cardiac surgery. The studies were conducted in Germany, Spain, France, Sweden, and Switzerland and included procedures such as CABG and other open cardiac surgeries. The mean age of participants was 65.2 years, and 68.4% were male. Risk of bias assessment using the Cochrane Risk of Bias 2 (ROB2) tool showed that two studies [2,6] had low risk, five studies [1,3,5,7,8] had some concerns, and one study [4] had high risk of bias (Tables 1-3).

**Table 1:** Summary of randomized controlled trials comparing sevoflurane and propofol for postoperative sedation after cardiac surgery This table summarizes the characteristics of randomized controlled trials included in the systematic review comparing inhaled sevoflurane with intravenous propofol for postoperative sedation in adult patients after cardiac surgery. The table includes study design details, recruitment period, country, eligibility criteria, type of cardiac surgery, intraoperative anesthetic regimen, duration, and method of postoperative sedation, sedation delivery devices, target sedation depth, duration of follow-up, and main study conclusions. Sedation was delivered using either inhalational sevoflurane (via AnaConDa or MIRUS systems) or intravenous propofol-based regimens, often in combination with opioids and/or neuromuscular blocking agents as per individual study protocols. Follow-up duration varied between studies and included ICU stay, hospital discharge, or up to 30-day postoperative outcomes, depending on the trial design. Abbreviations: ACD, Anaesthetic Conserving Device; ASA, American Society of Anesthesiologists physical status classification; BIS, bispectral index; CABG, coronary artery bypass grafting; CCS, Canadian Cardiovascular Society; CNS, central nervous system; COPD, chronic obstructive pulmonary disease; CPB, cardiopulmonary bypass; cTnI/TnI, cardiac troponin I; ECC, extracorporeal circulation; EF, ejection fraction; GDF-15, growth differentiation factor 15; ICU, intensive care unit; MAAS, Motor Activity Assessment Scale; MH, malignant hyperthermia; MI, myocardial infarction; NA, not available; RASS, Richmond Agitation-Sedation Scale

Study ID	Country	Inclusion Criteria	Exclusion Criteria	Type of Surgery	Intraoperative Anesthesia	Sedation Duration	Delivery Device	Sedation Target	Follow-Up	Conclusion
Steurer et al. [1]	Switzerland	Elective cardiac surgery with ECC	EF <30%, recent MI, emergency surgery, severe comorbidities	Valve surgery or CABG (on-pump)	Propofol, fentanyl, remifentanil	≥4 hours	AnaConDa	Titrated sedation	Until discharge	No significant difference in myocardial injury outcomes between groups.
Soro et al. [2]	Spain	Elective CABG requiring ≥4 h sedation	Combined surgery, organ dysfunction, and off-pump surgery	Elective CABG (on-pump)	Midazolam, etomidate, fentanyl, cisatracurium, propofol or sevoflurane	≥4 hours	AnaConDa	BIS 55-65 or RASS -2 to -3	72 hours	No significant differences in myocardial injury between groups.
Röhm et al. [3]	Germany	Elective CABG, age 18-80, ASA I-III	Severe cardiac, renal, respiratory, and hepatic disease	Elective CABG (on-pump)	Sufentanil, midazolam, pancuronium, sevoflurane	Until extubation (~8 h)	ACD	RASS -4 to -3, BIS 55-70	Until discharge	Sevoflurane is a safe alternative to propofol with faster extubation.
Marcos-Vidal et al. [4]	Spain	Age >18 years, coronary or mixed surgery with CPB	MH, propofol allergy, off-pump surgery, renal impairment	CABG or combined (on-pump)	Midazolam, fentanyl, etomidate, sevoflurane, remifentanil	≥120 minutes	AnaConDa	BIS 60-80	30 days	Sevoflurane is a valid alternative to propofol with no increase in renal injury.
Hellström et al. [5]	Sweden	Elective or subacute CABG	Off-pump CABG, additional surgery, circulatory support	Isolated CABG (on-pump)	Midazolam, fentanyl, propofol, sevoflurane, atracurium	≥2 hours until extubation criteria met	AnaConDa	MAAS 2-3	30 days	Sevoflurane leads to faster awakening and cooperation vs propofol.
Guinot et al. [6]	France	Age ≥18 years, cardiac surgery with CPB (CABG, valve, or combined)	Recent MI, chronic renal failure, dialysis, pregnancy	CABG, aortic valve, or combined (on-pump)	Propofol or sevoflurane, sufentanil, cisatracurium	Until haemodynamic stability and normothermia	MIRUS	RASS -2 to -3	30 days	Sevoflurane was not associated with reduced myocardial injury but showed higher acute renal failure rates.
Guerrero Orriach et al. [7]	Spain	Off-pump CABG, EuroSCORE <7	High surgical risk, instability, emergency surgery	Off-pump CABG	Etomidate, fentanyl, cisatracurium, sevoflurane or propofol, remifentanil	6 hours	AnaConDa	BIS 60-70	48 hours (30-day mortality noted)	Sevoflurane reduces myocardial injury markers compared with propofol.
Flinspach et al. [8]	Germany	Age >18 years, ICU admission following heart valve surgery, prior informed consent	Intolerance to volatile anesthetics (e.g., MH), severe COPD, major aortic surgery, and unexpected severe complications	Open heart valve surgery (mitral, tricuspid, aortic, combined)	Propofol, fentanyl, remifentanil	Discontinued 60 min after admission	AnaConDa and MIRUS	RASS -3 to -4	Until hospital discharge (delirium assessed up to Day 3)	Volatile sedation enables accelerated recovery and is a safe alternative in this population.

**Table 2:** Baseline characteristics of patients in randomized controlled trials comparing sevoflurane and propofol for postoperative cardiac surgery sedation This table summarizes baseline demographic, clinical, and intraoperative characteristics of patients included in randomized controlled trials comparing inhaled sevoflurane with intravenous propofol for postoperative sedation after cardiac surgery. Data are presented separately for sevoflurane and propofol groups within each study and include patient demographics (age, sex, height, body mass index), preoperative cardiac function (ejection fraction), operative risk scores (EuroSCORE and EuroSCORE II), and intraoperative variables such as cardiopulmonary bypass time, aortic cross-clamp time, number of bypass grafts, and total anesthesia time. Comorbidities, including diabetes mellitus and hypertension, are also reported where available. Values are presented as mean (standard deviation) unless otherwise indicated. Some variables are reported as ranges instead of mean (standard deviation), as originally provided in the included studies. Abbreviations: ACC, aortic cross-clamp time; BMI, body mass index; CPB, cardiopulmonary bypass; EuroSCORE, European System for Cardiac Operative Risk Evaluation; NA, not available; n, number of patients; SD, standard deviation

Study ID	Group (n)	Age (years), Mean (SD)	Male, n (%)	Height (cm), Mean (SD)	BMI (kg/m²), Mean (SD)	Ejection Fraction (%), Mean (SD)	EuroSCORE, Mean (SD)	EuroSCORE II, Mean (SD)	CPB Time (min), Mean (SD)	ACC Time (min), Mean (SD)	Number of Bypasses/Grafts, Mean (SD)	Anesthesia Time (min), Mean (SD)	Diabetes, n (%)	Hypertension, n (%)
Steurer et al. [1]	Sevoflurane (46)	63 (12.4)	32 (69)	NA	26.6 (3.7)	NA	NA	NA	141 (39)	92 (31)	NA	NA	NA	NA
	Propofol (56)	64 (14.7)	38 (67)	NA	27.1 (3.8)	NA	NA	NA	152 (49)	101 (34)	NA	NA	NA	NA
Soro et al. [2]	Sevoflurane (36)	68.3 (10.7)	27 (75.0)	164.4 (8.1)	27.4 (4.4)	57.4 (11.9)	4.1 (2.0)	NA	61.7 (16.2)	47.9 (15.7)	2.9 (0.7)	266.9 (36.5)	15 (41.7)	27 (75.0)
	Propofol (37)	69.4 (9.3)	30 (81.1)	165.2 (8.3)	28.9 (3.9)	58.1 (14.1)	4.1 (2.0)	NA	65.7 (18.1)	48.8 (15.5)	2.7 (0.8)	268.4 (49.5)	17 (45.9)	32 (86.5)
Röhm et al. [3]	Sevoflurane (35)	64.6 (8.6)	28 (80)	171.7 (8.7)	NA	58 (11)	NA	NA	68 (26)	41 (18)	NA	NA	NA	NA
	Propofol (35)	66.4 (8.0)	25 (78.1)	169.5 (10.2)	NA	57 (13)	NA	NA	61 (22)	37 (10)	NA	NA	NA	NA
Marcos-Vidal et al. [4]	Sevoflurane (67)	69.13 (10.52)	52 (77.6)	NA	28.05 (3.46)	NA	NA	6.17 (4.45)	124.04 (37.15)	NA	NA	366.28 (79.14)	NA	NA
	Propofol (62)	69.24 (11.85)	42 (67.7)	NA	27.70 (3.78)	NA	NA	6.91 (5.71)	117.47 (33.78)	NA	NA	338.98 (87.19)	NA	NA
Hellström et al. [5]	Sevoflurane (49)	65 (8)	NA	NA	28.8 (2)	NA	3.8 (2.1)	NA	62 (25)	NA	NA	206 (39)	NA	NA
	Propofol (50)	66 (11)	NA	NA	27.3 (4.6)	NA	4.3 (2.8)	NA	70 (19)	NA	NA	212 (48)	NA	NA
Guinot et al. [6]	Sevoflurane (42)	69 (10)	28 (60)	NA	28 (4)	61 (11)	NA	1.53 (1.1)	94.3 (28.4)	68.6 (27.63)	4 (1)	NA	15 (36)	32 (76)
	Propofol (39)	68 (11)	24 (67)	NA	28 (5)	62 (7)	NA	1.25 (0.66)	88.3 (38.48)	67.3 (20)	4 (1)	NA	15 (38)	32 (80)
Guerrero Orriach et al. [7]	Sevoflurane (20)	61-73	9 (45)	162-171	NA	61-65	4.75 (0.8)	NA	NA	NA	2 (0.53)	265 (54)	NA	NA
	Propofol (20)	62-74	10 (50)	160-173	NA	62-67	4 (0.53)	NA	NA	NA	2 (0.53)	254 (64)	NA	NA
Flinspach et al. [8]	Sevoflurane (47)	60 (13.76)	33 (70.2)	NA	26.23 (2)	NA	NA	1.26 (0.81)	98.33 (24.47)	62 (15.29)	NA	290 (64.23)	9 (19.1)	36 (76.6)
	Propofol (47)	64 (10.7)	33 (70.2)	NA	25.56 (2.82)	NA	NA	1.43 (0.95)	109 (43.59)	63.3 (16.82)	NA	306 (76.47)	6 (12.8)	34 (72.3)

**Table 3:** Risk of bias 2 assessment of included randomized controlled trials Risk of Bias 2 (RoB 2) assessment of included randomized controlled trials evaluating volatile anesthetics versus intravenous or alternative sedation strategies in cardiac surgery and postoperative intensive care settings [12]. Domains are based on the Cochrane RoB 2 tool: D1 = bias arising from the randomization process; D2 = bias due to deviations from intended interventions; D3 = bias due to missing outcome data; D4 = bias in measurement of the outcome; and D5 = bias in selection of the reported result. Each domain is rated as “Low risk,” “Some concerns,” or “High risk,” according to RoB 2 signalling questions. The overall risk of bias reflects the highest level of concern across domains, as per RoB 2 guidance. Study identifiers are presented as the first author followed by the reference number corresponding to the reference list.

Study	D1: Randomization Process	D2: Deviations From Intended Interventions	D3: Missing Outcome Data	D4: Measurement of the Outcome	D5: Selection of Reported Results	Overall Risk of Bias
Steurer et al. [1]	Low	Some concerns	Low	Some concerns	Low	Some concerns
Soro et al. [2]	Low	Low	Low	Low	Low	Low
Röhm et al. [3]	Low	Low	Low	Some concerns	Low	Some concerns
Marcos-Vidal et al. [4]	High	High	Low	Low	Some concerns	High
Hellström et al. [5]	Low	Some concerns	Low	Some concerns	Low	Some concerns
Guinot et al. [6]	Low	Low	Low	Low	Low	Low
Guerrero Orriach et al. [7]	Some concerns	Some concerns	Low	Low	Some concerns	Some concerns
Flinspach et al. [8]	Low	Some concerns	Low	Some concerns	Low	Some concerns

Primary Outcomes

Four studies (n = 344) [1,2,4,6] reported time to extubation. There was no significant difference between sevoflurane and propofol (MD -62.67, 95% CI -157.23 to 31.90; p = 0.2977), with high heterogeneity (I² = 91.7%). Sensitivity analysis, excluding one study [4], resulted in a significant reduction in extubation time favoring sevoflurane (MD -72.51, 95% CI -135.83 to -9.20; p = 0.0388), with moderate heterogeneity (I² = 54.8%). Subgroup analysis suggested a benefit of sevoflurane in mixed and valve surgery populations [2,6], while no significant difference was observed in isolated CABG patients [1,4].

Five studies (n = 452) [1,2,3,5,7] reported ICU LOS. No significant difference was found between groups (MD -0.29, 95% CI -4.10 to 3.51), with low heterogeneity (I² = 27.1%). Sensitivity and subgroup analyses did not change these results.

Four studies (n = 323) [2,3,6,8] reported hospital LOS. There was no significant difference between sevoflurane and propofol (MD -0.75, 95% CI -3.13 to 1.63), with low heterogeneity (I² = 35.8%). Sensitivity and subgroup analyses confirmed result stability.

Secondary Outcomes

No significant differences were observed between groups for any secondary outcomes. Four studies [1,3,5,7] reported mortality, showing an OR of 2.50 (95% CI 0.77 to 8.19), with no heterogeneity (I² = 0%). Three studies [2,4,6] reported ARF, showing an OR of 0.95 (95% CI 0.15 to 6.10), with moderate heterogeneity (I² = 48.7%). Three studies [1,6,8] reported PONV, showing an OR of 0.84 (95% CI 0.24 to 2.90), with low heterogeneity (I² = 24.4%). Three studies [2,3,5] reported atrial fibrillation, showing an OR of 1.09 (95% CI 0.41 to 2.88), with no heterogeneity (I² = 0%).

Summary of Findings

The main outcomes are summarized in Table 4. No significant differences were observed between sevoflurane and propofol for ICU or hospital LOS. Time to extubation showed no overall difference; however, sensitivity analysis suggested a shorter extubation time with sevoflurane. Secondary outcomes, including mortality, ARF, atrial fibrillation, and PONV, were comparable between groups.

**Table 4:** Summary of findings: sevoflurane vs propofol for postoperative sedation after cardiac surgery This table summarizes the pooled effect estimates from randomized controlled trials comparing inhaled sevoflurane with intravenous propofol for postoperative sedation in adult patients following cardiac surgery. Outcomes are presented as mean differences (MD) for continuous variables and odds ratios (OR) for dichotomous variables, each with corresponding 95% confidence intervals (CI). Negative MD values favor sevoflurane for time-based outcomes (e.g., shorter time to extubation or length of stay). For dichotomous outcomes, an OR <1 favors sevoflurane, whereas an OR >1 favors propofol. Heterogeneity across studies was assessed using the I² statistic, with values >60% indicating substantial heterogeneity. Sensitivity analysis was performed where indicated to assess the robustness of results. Subgroup analyses were conducted based on type of cardiac surgery. The certainty of evidence was assessed qualitatively based on study limitations (risk of bias), inconsistency, imprecision, and potential publication bias. Abbreviations: MD, mean difference; OR, odds ratio; CI, confidence interval; ICU, intensive care unit; LOS, length of stay.

Outcome		No. of Studies (n)	Total Patients	Effect Estimate (95% CI)	Heterogeneity (I²)	Interpretation	Certainty of Evidence
Primary Outcomes	Time to extubation	4	344	MD -62.67 min (-157.23 to 31.90)	0.917	No significant difference	Low
	Time to extubation (sensitivity analysis)	3	-	MD -72.51 min (-135.83 to -9.20)	0.548	Favors sevoflurane	Low
	ICU length of stay	5	452	MD -0.29 days (-4.10 to 3.51)	0.271	No significant difference	Moderate
	Hospital length of stay	4	323	MD -0.75 days (-3.13 to 1.63)	0.358	No significant difference	Moderate
Secondary Outcomes	Mortality	4	-	OR 2.50 (0.77 to 8.19)	0	No significant difference	Low
	Acute renal failure	3	-	OR 0.95 (0.15 to 6.10)	0.487	No significant difference	Low
	Atrial fibrillation	3	-	OR 1.09 (0.41 to 2.88)	0	No significant difference	Low
	Postoperative nausea & vomiting	3	-	OR 0.84 (0.24 to 2.90)	0.244	No significant difference	Low

Limitations

This study has several limitations. The number of included RCTs was small, with limited sample sizes for key outcomes such as mortality, ARF, and atrial fibrillation. Substantial clinical heterogeneity was present across studies, including differences in surgical procedures, cardiopulmonary bypass exposure, sedation protocols, extubation criteria, and delivery devices. Evidence of potential publication bias and the sensitivity of results to study exclusion further reduces the certainty of the findings. Overall, larger and more standardized trials are needed to confirm these results.

## Conclusions

Sevoflurane may serve as a recovery-enhancing sedative that can shorten the time to extubation after cardiac surgery. However, this potential benefit does not appear to translate into consistent improvements in ICU or hospital LOS or in postoperative complication rates. Overall, sevoflurane represents a reasonable alternative to propofol in selected settings, but it cannot be considered a clearly superior standard approach. Further well-designed, large-scale studies are needed to clarify its clinical impact.

## References

[1] Steurer MP, Steurer MA, Baulig W (2012). Late pharmacologic conditioning with volatile anesthetics after cardiac surgery. Crit Care.

[2] Soro M, Gallego L, Silva V (2012). Cardioprotective effect of sevoflurane and propofol during anaesthesia and the postoperative period in coronary bypass graft surgery: a double-blind randomised study. Eur J Anaesthesiol.

[3] Röhm KD, Wolf MW, Schöllhorn T, Schellhaass A, Boldt J, Piper SN (2008). Short-term sevoflurane sedation using the anaesthetic conserving device after cardiothoracic surgery. Intensive Care Med.

[4] Marcos-Vidal JM, González R, Garcia C, Soria C, Galiana M, De Prada B (2014). Sedation with sevoflurane in postoperative cardiac surgery: influence on troponin T and creatinine values. Heart Lung Vessel.

[5] Hellström J, Öwall A, Bergström J, Sackey PV (2011). Cardiac outcome after sevoflurane versus propofol sedation following coronary bypass surgery: a pilot study. Acta Anaesthesiol Scand.

[6] Guinot PG, Ellouze O, Grosjean S (2020). Anaesthesia and ICU sedation with sevoflurane do not reduce myocardial injury in patients undergoing cardiac surgery: a randomized prospective study. Medicine (Baltimore).

[7] Guerrero Orriach JL, Galán Ortega M, Ramirez Aliaga M, Iglesias P, Rubio Navarro M, Cruz Mañas J (2013). Prolonged sevoflurane administration in the off-pump coronary artery bypass graft surgery: beneficial effects. J Crit Care.

[8] Flinspach AN, Raimann FJ, Kaiser P, Pfaff M, Zacharowski K, Neef V, Adam EH (2024). Volatile versus propofol sedation after cardiac valve surgery: a single-center prospective randomized controlled trial. Crit Care.

[9] Page MJ, McKenzie JE, Bossuyt PM (2021). The PRISMA 2020 statement: an updated guideline for reporting systematic reviews. Rev Esp Cardiol (Engl Ed).

[10] Schardt C, Adams MB, Owens T, Keitz S, Fontelo P (2007). Utilization of the PICO framework to improve searching PubMed for clinical questions. BMC Med Inform Decis Mak.

[11] Sessler CN, Gosnell MS, Grap MJ (2002). The Richmond Agitation-Sedation Scale: validity and reliability in adult intensive care unit patients. Am J Respir Crit Care Med.

[12] Crocker TF, Lam N, Jordão M (2023). Risk-of-bias assessment using Cochrane's revised tool for randomized trials (RoB 2) was useful but challenging and resource-intensive: observations from a systematic review. J Clin Epidemiol.

